# Structure Determination of *β*-Glucans from * Ganoderma lucidum* with Matrix-assisted Laser Desorption/ionization (MALDI) Mass Spectrometry

**DOI:** 10.3390/molecules13081538

**Published:** 2008-08-03

**Authors:** Wei-Ting Hung, Shwu-Huey Wang, Chung-Hsuan Chen, Wen-Bin Yang

**Affiliations:** 1 Genomics Research Center, Academia Sinica, No. 128, Academia Road Section 2, Nan-Kang, Taipei 11529, Taiwan; 2 Instrument Center, Taipei Medical University, Taipei 11031, Taiwan

**Keywords:** Polysaccharides, matrix-assisted laser desorption/ionization (MALDI), *Ganoderma lucidum*, β-glucan, 2,5-dihydroxybenzoic acid (2,5-DHB).

## Abstract

A novel method that uses matrix-assisted laser desorption/ionization (MALDI) mass spectrometry to analyze molecular weight and sequencing of glucan in *Ganoderma lucidum* is presented. Thus, *β*-glucan, which was isolated from fruiting bodies of *G. lucidum*, was measured in a direct and fast way using MALDI mass spectrometry. In addition, tandem mass spectrometry of permethylated glucans of *G. lucidum*, dextran, curdlan and maltohexaose were also pursued and different fragment patterns were obtained. The *G. lucidum* glucan structure was determined and this method for linkage analysis of permethylated glucan has been proven feasible.

## Introduction

*Ganoderma* species (a group of medicinal fungi), including *Ganoderma lucidum* (Reishi in Japan or Ling-Zhi in China), has been used for a long time in China to prevent and treat various human diseases. Matrix-assisted laser desorption/ionization (MALDI) has been developed as a successful tool for the analysis of biopolymers and macromolecules [1,2,3]. The ions produced by MALDI are predominantly singly charged molecular ions so that simple mass spectra can be used for distribution analysis of neutral polysaccharides. In recent years, several mass spectrometric studies have been focused on the analysis of polysaccharide materials or oligosaccharides [4,5,6]. Direct detection of saccharides in sulfuric acid/phenol has been commonly used before, but it can not derive information on molecular weights of different saccharides and the differences among pentoses, hexoses and unusual sugars. Gel permeation chromatography (GPC) has been used for measurements of the size of polysaccharides by comparison of results with appropriate polysaccharide standards [7]. Nevertheless, the resolution of GPC is generally poor and the process is very time consuming. Since most polysaccharides are polydispersable to molecular weight, GPC is not a direct method for accurate mass measurements. On the other hand, MALDI-MS is practical for more qualitative and mass determination. In our previous study [8,9], we found that non-derivatized and derivatized polysaccharides were successfully analyzed by a straightforward MALDI mass spectrometry when 2’,4’,6’-trihydroxyacetophenone (THAP) or 2,5-dihydroxybenzoic acid (2,5-DHB) was used as matrix. In this work, we measured both non-derivatized and derivatized glucans from *G. lucidum* polysaccharides. In addition, comparisons of molecular weight and structural information are made between the *G. lucidum* polysaccharides and glucan standards from dextran, curdlan and starch using MALDI-TOF (time-of-flight) mass spectrometry. The linkage information of the permethylated oligomer ions from *G. lucidum* was established through the use of on-line MS/MS experiments.

Polysaccharides extracted from *G. lucidum* (also known as Ling-Zhi) have been widely used and studied for their immunomodulating and anti-cancer potential [10,11,12,13,14]. The main components of *G. lucidum* polysaccharides are (1→3) and (1→6)-*β*-d-glucan. *β*-d-Glucan is a carbohydrate polymer with chains of glucose molecules linked together by *β*-glycosidic linkages [15,16,17]. Up to now, *β*-glucan isolated from *G. lucidum* has not been studied using the MALDI-MS method. In this work, we have analyzed not only non-derivatized *G. lucidum* polysaccharides, but also the permethylated derivatized *β*-glucan.

## Results and Discussion

**Figure 1:**
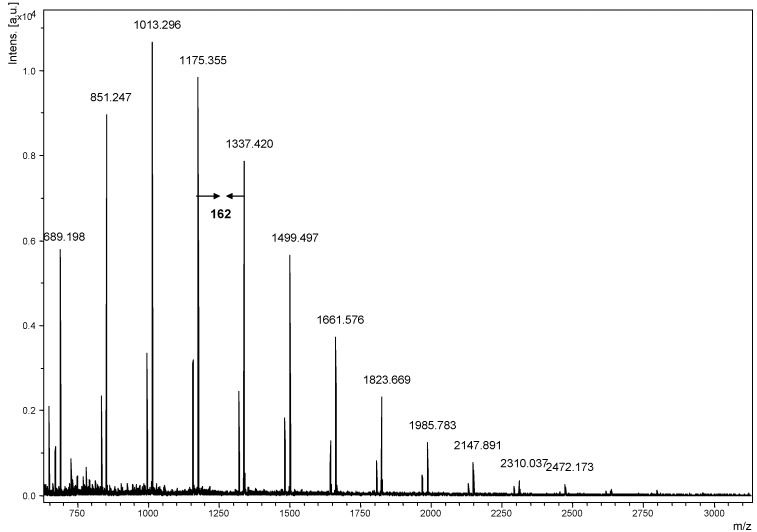
MALDI-TOF mass spectrum of *Ganoderma lucidum* glucans using 2,5-DHB as matrix. The mass difference of 162.1 Da between neighboring peaks is observed.

Our goal in this study was to examine the structure of glucans from *G. lucidum.* The ground fruiting body of *G. lucidum* was extracted by hot water to release polysaccharides and the crude polysaccharides were further hydrolyzed by a mild acid degradation (2M TFA, 50 ^o^C, 2 hrs) to obtain a low molecular weight and water soluble polysaccharide fraction. In order to investigate the sugar composition of *G. lucidum* polysaccharide, this polysaccharide fraction was hydrolyzed into monosaccharides by acidic hydrolysis (4M TFA, 121 ^o^C, 6 hrs) and high performance anion exchange chromatography with pulsed amperometric detection (HPAEC-PAD) was used to measure its sugar composition. We found only glucose present in this fraction of *G. lucidum*. By NMR analysis, we found that these novel glucans have a *β*-(1→3)/*β*-(1→6)-configuration at the glycosidic bonds (data not shown). This result is in agreement with the previous research in which polysaccharides are isolated from *G. lucidum* [18,19]. However, here we used MALDI-TOF mass spectrometry to determine *G. lucidum* glucan. For the analysis of native polysaccharides in our previous work [8,9] we observed THAP and 2,5-DHB are good matrices that substantially enhanced signals for native pullulans standard in MALDI mass spectrometry. In this study we also use both matrices for the mass spectrometry analysis. Both positive and negative ions of polysaccharides were obtained. In this study, neutral carbohydrates were found to prefer the positive ion mode, due to the attachment of alkali metal ions. The *G. lucidum* glucans were observed as sodiated ions ([Glcn + Na]^+^) and the corresponding mass spectrum is given in Figure 1. The mass of the singly charged molecular ion were calculated as 162.14n + 22.990 Da and 162.14n + 22.990 + 18.015 (mass of reducing end residue) Da respectively, where n is the number of glucose units. For example, if n = 10, the mass of the corresponding glucan should be 1662.4 Da respectively. The mass region of ion peaks was observed with a peak-to-peak mass difference of 162.1 Da, consistent with the repeating unit of the β-(1→3)-glucan. Similar observations have been reported previously [7,20,21] and were attributed to the fragmentation of the high mass polysaccharides. The MALDI-TOF mass spectrum in Figure 1 showed that *G. lucidum* glucans are composed of a mixture of glucose polymers with molecular weights around 689.2 ~ 2634.2 Da. This is the first report on measuring the glucan having DP = 4 ~ 16 in *G. lucidum* with mass spectrometry.

**Figure 2:**
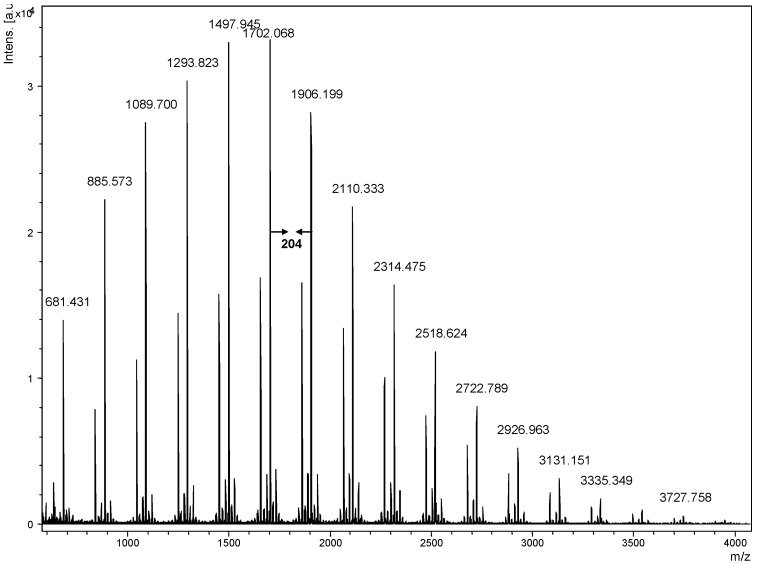
MALDI-TOF mass spectrum of permethylated *G. lucidum* glucans with 2,5-DHB as matrix. A peak-to-peak mass difference of 204.1 Da is observed.

The known glucans of different molecular weights such as dextran (1→6), amylose (1→4) and curdlan (1→3) have been analyzed by MALDI for molecular weight determination [20,21,22,23]. However, up to now the chemical linkages between these glucans still have not been determined by mass spectrometry. In this work, we determined the sequences of oligosaccharides and polysaccharides. We prepared the glucan samples by water extraction and a size exclusion chromatographic method to obtain low molecular weight oligosaccharides as well as polysaccharides and these glucans were derivatized by permethylation. Permethylation produced stronger sodium attached ions than non-methylated glucans for maltohexose, dextran, curdlan and *G. lucidum*. In Figure 2, we measured permethylated *G. lucidum* glucans using 2,5-DHB as a matrix. The *G. lucidum* glucans were observed as sodium attached ions and molecular masses were calculated as 219.13 (a terminal sugar) + 204.13n + 22.99 Da and 219.13 + 204.13n + 31.02 (mass of reducing end residue) + 22.99 Da, respectively, where n is the number of 2,4,6-trimethyl glucosyl unit. For example, if a decasaccharide, the mass of the corresponding glucan should be 2079.3 Da ([2,3,4,6-tetramethylglucosyl-(2,4,6-trimethyl glucosyl)_9_ + Na]+) and 2110.3 Da ([2,3,4,6-tetramethylglucosyl-(2,4,6-trimethyl glucosyl)_9_ + 31.02 + Na]+) respectively. The mass region of ion peaks was observed with a peak-to-peak mass difference of 204.1 Da consistent with the repeating unit of the permethylated β-glucan from 681.4 ~ 3727.8 Da.

**Figure 3:**
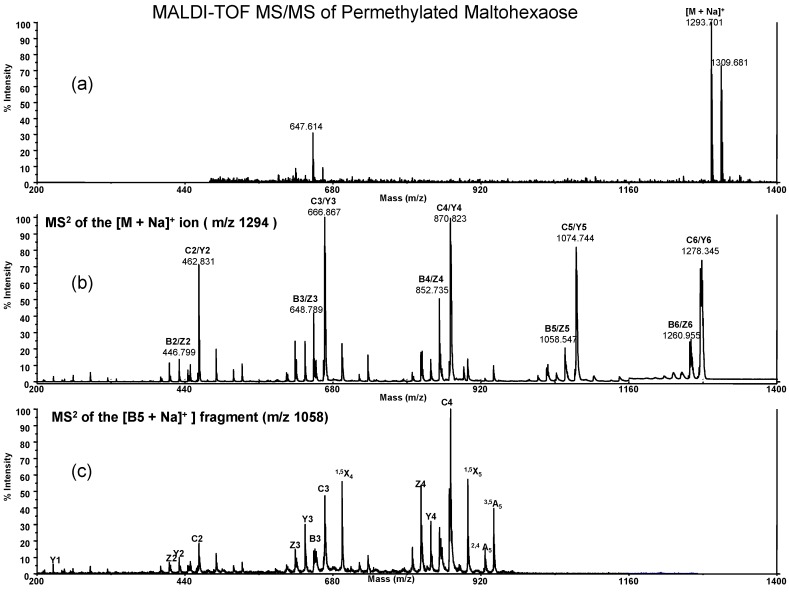
MALDI-TOF MS/MS of permethylated maltohexaose in 2,5-DHB as matrix. The ion of maltohexaose (*m/z* = 1293.8, [M + Na]^+^) shown in figure 3a. MS^2^ at the permethylated maltohexaose ion ([M + Na]^+^, *m/z* = 1293.8) shown in 3b and MS^2^ at the B_5_ sodiated fragment ion (*m/z* = 1058.8) shown in figure 3c. The major cross-ring fragment ions at B_5_~C_4_ region (*m/z* = 1058.8 ~ 870.8) are 941.0 (^3,5A^_5_), 927.0 (^2,4A^_5_) and 898.9 (^1,5^X_5_).

In order to obtain sequencing information about *G. lucidum* glucans we used the tandem mass (MS/MS) method to get linkage information. MS^2^ spectra for various oligosaccharides such as dextran (1→6), oligomaltose (1→4), curdlan (1→3) and *G. lucidum* polysaccharides (1→3/1→6) were obtained to show different fragmented ions for linkage assignment. In the past, Creaser *et al.* [24] published the structural analysis of non-derivative maltoheptaose using the MALDI-TOF MS^n^ method. They obtained a strong sodium attached singly charged ion peak at *m/z* 1175.3, which was subsequently analyzed by a tandem mass spectrometry to yield a number of fragment ions. Here, we investigated MALDI of permethylated maltohexaose and the peak showed at *m/z* 1293.7 as [M + Na]^+^ ion (Figure 3a). The MS/MS spectra of the [M + Na]^+^ ion of permethylated maltohexaose at *m/z* 1293.7 are shown in Figure 3b. The spectra were dominated by peaks resulting from cleavage at glycosidic bonds, giving the C/Y ion series (*m/z* 1278.3, 1074.7, 870.8, 666.9, 462.8) and a less intense series of B/Z ions with 18 Da lower (*m/z* 1260.9, 1058.8, 852.7, 648.8, 446.8), respectively. The nomenclatures for the fragment ions were introduced by Domon and Costello [25]. Although reducing terminal ions (C ions) are often more abundant, it is the B ions that are more informative when probed by MS/MS analysis. Pyranose unsaturation (B ions) coupled with metal ion chelation appears to lower energy barriers to ring rupture via a classical retro Diels-Alder decomposition reaction to give cross-ring cleavages (A-type or X-type fragments) [26,27]. Figure 3c showed the MS^2^ of the B_5_ ion, with *m/z* at 1058.8 and the major cross-ring fragment ions at *m/z* 1058.8 ~ 870.8 (B_5_~C_4_ region) are 941.0 (^3,5A^_5_), 927.0 (^2,4A^_5_) and 898.9 (^1,5^X_5_), respectively. We found a high intensity of ^3,5A^_5_ ion to indicate a 1→4 linkage while 1→3 linkage is absent. The ^2,4A^_5_ ion is present of only for the 1→4 or 1→3 linkage but not in 1→6 linkage and ^1,5^X_5_ fragment ions are X type ion with the similar ions also found for ^1,5^X_4, _^1,5^X_3_ which also appear in MS/MS spectrum of permethylated maltohexaose at *m/z* 1293.7 (figure 3b). This method got a similar mode of fragment ions when probed B ions at B_6_, B_4_, B_3_ and B_2_ by MS^2^ analysis. Although the fragment ions from B_5_ ion still have not enough information for identification of linkage analysis between this region of ring fragments, its spectrum is quite different and can be used for the comparison of various glucans.

**Figure 4:**
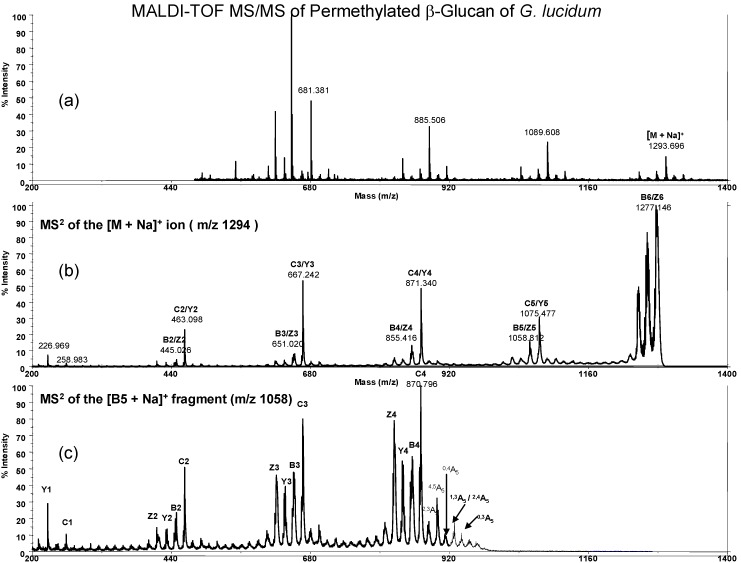
MALDI-TOF MS/MS of permethylated *G. lucidum* glucans. The ion of permethylated *G. lucidum* hexasaccharide (*m/z* = 1293.7, [M + Na]^+^) shown in figure 4a. MS^2^ at the hexasaccharide ion ([M + Na]^+^, *m/z* = 1293.8) shown in 4b and MS^2^ at the B_5_ sodiated fragment ion (*m/z* = 1058.8) shown in figure 4c. The major cross-ring fragment ions at B_5_~C_4_ region are 940.7 (^0,3A^_5_), 928.7 (^1,3A^_5_/^2,4A^_5_), 912.3 (^0,4A^_5_), 898.7 (^1,5^X_5_/^4,5A^_5_) and 883.1 (^2,3A^_5_), respectively.

Here we also measured permethylated *G. lucidum* glucan, which is derived from acidic degradation (Figure 4a). And MS^2^ at *m/z* = 1293.7 of this permethylated *G. lucidum* hexasaccharide (Figure 4b) was dominated by peaks resulting from cleavage at glycosidic bonds, giving the C/Y ion series and a less intense series of B/Z ions, respectively, which are same as the one mentioned in the above (Figure 3b). The MS^2^ spectrum of permethylated *G. lucidum* glucan B_5_ ion at *m/z* 1058.8 is shown in Figure 4c. The major fragment ions are the Y and C ions (1277.1, 1075.5, 871.3, 667.2, 463.1 *m/z*), as usual. However, the B_5_ ions differ between the permethylated *G. lucidum* hexasaccharide (Figure 4c) and permethylated maltohexaose (Figure 3c). The fragmented ions from *m/z* at 1058.8 ~ 871.3 are 940.7 (^0,3A^_5_), 928.7 (^1,3A^_5_/^2,4A^_5_), 912.3 (^0,4A^_5_), 898.7 (^1,5^X_5_) and 883.1 (^2,3A^_5_), respectively. For the ions with m/z at 912.3 (^0,4A^_5_) and 898.7 (^4,5A^_5_), it indicates that *G. lucidum* glucan has 1→6 linkage and 883.1 (^2,3A^_5_) indicates out the presence of 1→3 linkage. From Figure 4, we confirm that this methylated glucan has 1→3 and 1→6 linkage between glycosidic bonds. 

**Figure 5:**
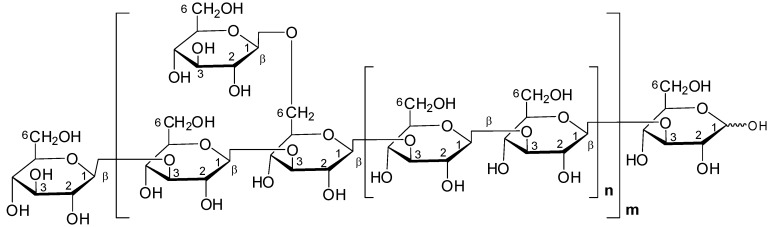
Possible repeating units of *G. lucidum* glucans.

**Figure 6:**
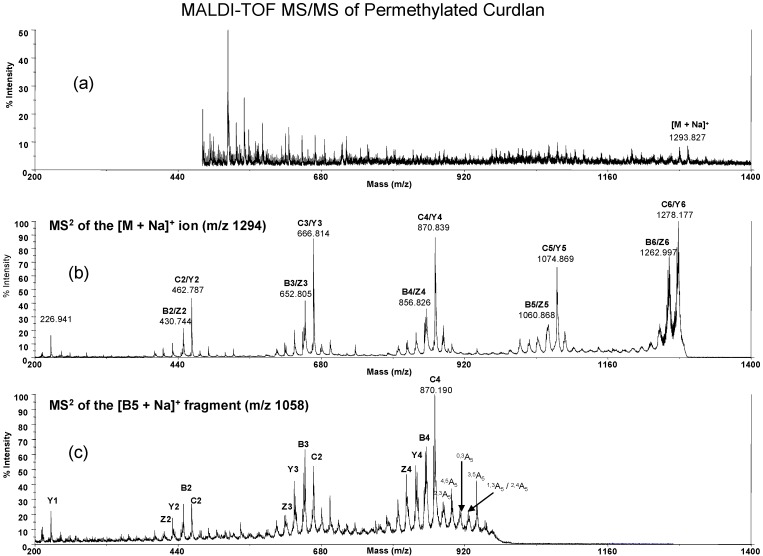
MALDI-TOF MS/MS of permethylated curdlan in 2,5-DHB as matrix. Shown are the hexasaccharide ion (*m/z* = 1293.8, [M + Na]^+^) (6a), MS^2^ at the hexasaccharide ion ([M + Na]^+^, *m/z* = 1293.8) (6b) and MS^2^ at the B_5_ sodiated fragment ion (*m/z* = 1058.8) (6c). The major cross-ring fragment ions at B_5_~C_4_ region are 941.0 (^0,3A^_5_), 927.9 (^1,3A^_5_/^2,4A^_5_), 911.0 (^0,4A^_5_), 898.9 (^1,5^X_5_) and 883.1 (^2,3A^_5_).

**Figure 7:**
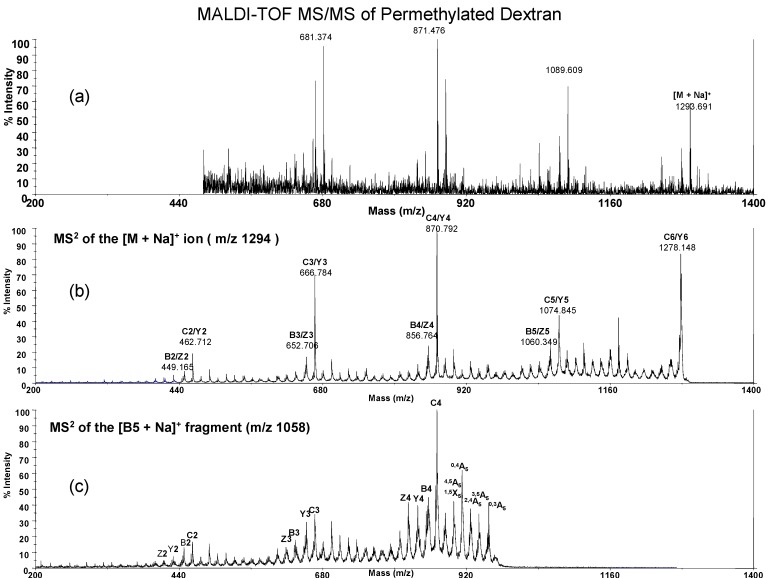
MALDI-TOF MS/MS of permethylated dextran glucans. The hexasaccharide ion (*m/z* = 1293.8, [M + Na]^+^) is shown in 7a. MS^2^ at the hexasaccharide ion ([M + Na]^+^, *m/z* = 1293.8) is shown in 7b and MS^2^ at the B_5_ sodiated fragment ion (*m/z* = 1058.8) is shown in 7c. The major cross-ring fragment ions at B_5_~C_4_ region are 957.3 (^0,3A^_5_), 940.8 (^3,5A^_5_), 925.9 (^1,4A^_5_), 912.9 (^0,4A^_5_) and 898.9 (^4,5A^_5_/^1,5^X_5_) respectively.

For comparison we also prepared permethylated low molecular weight glucan from curdlan (Figure 6) and dextran (Figure 7). The MS/MS spectrum as its B_5_ ion (*m/z* 1058.8) also gave the cross-ring fragment ions for glucan structural determination. In brief, permethylated *G. lucidum* glucan displayed similar cross-ring fragment ions with curdlan at 940.7 (^0,3A^_5_), 928.7 (^1,3A^_5_/^2,4A^_5_), 912.3 (^0,4A^_5_), 898.7 (^1,5^X_5_/^4,5A^_5_) and 883.1 (^2,3A^_5_) and *G. lucidum* glucan also has a weaker signal at 898.7 m/z (^4,5A^_5_) that is the same as dextran ^4,5A^_5_ ion. In this study we did not obtain the ratio of 1→3/1→6 linkage of *G. lucidum* glucan. However, the real glucan structure in *G. lucidum* contains trace amount of 1→6 side chains, which were previously identified by NMR and GC/MS analysis. The mass spectrometric study for quantification of branched glucan still faces challenges.

Here we focus on B type ions for linkage analysis, since the sugar ring cleavage is easier so that fragment ions are strong enough for the analysis. The ions from permethylated hexasaccharide (*m/z* = 1293.7) to disaccharide (*m/z* = 446.7) are examined for the study of different fragmented ions on these glucans. Based on the MALDI-TOF mass analysis we determined not only the molecular weights of glucans, but also the sugar sequences. The results shown that *G. lucidum* glucan has a branched (1→3/1→6)-glucan linkage structure and the proposed chemical structure of *G. lucidum* glucan is as shown in Figure 5.

## Conclusions

The use of MALDI-TOF mass spectrometry for analyzing polysaccharides of *G. lucidum* is successfully demonstrated. The results showed that *G. lucidum* has an abundance of glucan and the chemical structure involves (1→3/1→6) linkages at the glycosidic bonds. This is the first time a detailed analysis of the molecular weight and glycosidic bond linkages *G. lucidum* polysaccharides. The method can be applied to sequencing of polysaccharides from other natural products or synthetic organic chemicals in the future.

## Experimental Section

### Materials

Matrices of 2’,4’,6’-trihydroxyacetophenone (THAP) and 2,5-dihydroxybenzoic acid (2,5-DHB) were purchased from Sigma Chem. Co. and methanol, dimethyl sulfoxide, acetonitrile, methyl iodide, sodium hydride and trifluoroacetic acid were purchased from Merck & Co., Inc. Glucan from dextran, curdlan, oligomaltose were obtained from Sigma Chem. Co. All chemicals were analytical grade and used without further purification and double distilled water was used throughout polysaccharide extraction and purification. Glucan from ground fruiting body of *G. lucidum* was extracted by hot water and TFA degradation.

### Instrumentation

MS spectra of the sodium adducts of oligosaccharides [M + Na]^+^ were acquired using an Ultraflex^TM^ TOF/TOF mass spectrometer (Bruker Daltonik GmbH, Bremen, Germany) with gridless ion optics under control of Flexcontrol 2.0. The instrument, equipped with the SCOUT^TM^ high-resolution optics with X-Y multisample probe and gridless reflector, was used at a maximum accelerating potential of 25 kV and was operated in reflector mode for MS analysis. Ionization was accomplished with a 337-nm beam from a nitrogen laser with a repetition rate of 20 Hz. The output signal from the detector was digitized at a sampling rate of 2 GHz. The high-performance anion-exchange chromatography with a pulsed amperometric detection (HPAEC-PAD) for sugar composition analysis was performed on a Dionex BioLC system (Dionex, Sunnyvale, CA, USA) using a CarboPac PA10 analytical column (2 mm × 250 mm) and a CarboPac PA10 guard column (2 mm × 50 mm). GC-MS system (Thermo Fisher Scientific, Inc, Waltham, MA 024253, USA) with PolarisQ ion trap and BPX-70 column were used for alditol acetates and partially methylated alditol acetates analysis. The NMR spectra were recorded on a Bruker 500 MHz NMR spectrometer (Bruker BioSpin GmbH, Rheinstetten, Germany) with 5 mm Cryoprobe DCI ^1^H/^13^C.

### Mass spectral analysis of glucans

For MALDI-MS analysis, the samples were prepared by standard dried-droplet preparation on stainless steel MALDI targets using 2,5-dihydroxybenzoic acid or 2’,4’,6’-trihydroxyacetophenone as matrix. The non-derivatized and derivatized (permethylation) oligo-, polysaccharides were mixed with 10 mM of 2,5-DHB or THAP in the ratio of 1:1. In MS mode, the spectra were accumulated in average of 2000–3000 shuts.

### Preparation of glucans

The dried fruiting bodies of *G. lucidum* were ground and extracted with dd-water (double-distilled water) at 100 ^o^C for 12 hr. The suspension was centrifuged (4000 rpm, 1 hr) to remove the insoluble materials, and the water-soluble crude polysaccharide was obtained (11%). The crude polysaccharide (0.5 g) was degraded into a low molecular weight glucan using a chemical method (2M TFA 50 ^o^C, 2h) and then purified through a gel-filtration chromatography using a Sephadex G-15 column with dd-water as the eluent to obtain a glucan fraction (15~20%). Other low molecular weight glucans from dextran and curdlan were obtained by the similar method for *G. lucidum* glucan.

### Composition analysis of G. lucidum glucan

The *G. lucidum* glucan (50 mg) was hydrolyzed with 4 M trifluoroacetic acid (TFA) at 121 ºC for 4 h. The mixture was cooled, and TFA was removed under reduced pressure. The acid hydrolysate was washed with 50% aqueous methanol, and then dried under vacuum. Reduction of monosaccharides in the hydrolysate was carried out by using NaBH_4_ (80 mg) in MeOH at room temperature for 30 min. This procedure was repeated for 3–5 times to assure complete reduction of monosaccharides to alditols. The mixture was washed with concentrated HCl and MeOH, and then dried under vacuum. The alditols reduction products were subjected to acetylation using acetic anhydride in pyridine (1:2) at 80 ºC for 2 h, followed by incubation at 25 ºC for 16 h. The alditol peracetates were extracted by chloroform/2 M HCl (2:1), and washed carefully with saturated NaHCO_3_. After removal of chloroform, the composition of *G. lucidum* glucan of alditol peracetates was determined by GC–MS analysis. In GC–MS the alditol acetates were separated by BPX-70 column (SGE Analytical Science Pty Ltd., Victoria 3134, Australia). Oven temperature was increased from 38 ºC to 150 ºC at 5 ºC per min, then to 230 ºC at 3 ºC per min and further increased to 260 ºC and kept there for 5 min with the carrier gas (He) kept at a flow rate of 1 mL/min. In HPAEC-PAD analysis, The *G. lucidum* glucan (50 mg) was hydrolyzed by 4 M TFA at 121 ºC for 4 h. TFA was removed under reduced pressure and the acid hydrolysate was determined by Dionex BioLC system. A CarboPac PA10 analytical column (2 × 250 nm) and a CarboPac PA10 guard column were used. Samples were eluted using a linear gradient of 16 mM NaOH over 20 min and flow rate was set as 0.25 mL/min. Determinations were performed by comparison of peak areas with monosaccharide standards. Analyses were performed twice for each sample.

### Linkage analysis of G. lucidum glucan

The *G. lucidum* glucan (30 mg) was dissolved in DMSO (2 mL), treated with NaH (30 mg, 60% in mineral oil) for 10 min with continuous stirring, and ice cold MeI (1 mL) was added. After stirring at room temperature for 12 h, the methylated glucan in suspension were separated by addition of chloroform/water (2:1). The organic phase was washed with 10% Na_2_S_2_O_3_ and water, and then concentrated under reduced pressure to give the crude product of methylated polysaccharides. According to the above-described procedure, the product of methylated glucan was similarly hydrolyzed with TFA, reduced with NaBH_4_, and acetylated with Ac_2_O/pyridine to give methylated alditol peracetates. The composition of methylated alditol peracetates were determined by GC–MS analysis.
